# Study on the clinical mechanism of Tong-Xie-An-Chang Decoction in the treatment of diarrheal irritable bowel syndrome based on single-cell sequencing technology

**DOI:** 10.1097/MD.0000000000023868

**Published:** 2020-12-24

**Authors:** Xiang Tan, Xing-jie Zhao, Jun-xiang Li, Chun-e Xie, Wen-jing Pei, Lei Shi, Fu-shun Kou, Ya-li Yuan, Xiao-xuan Xue

**Affiliations:** a Grauate school, Beijing University of Chinese Medicine, No. 11, North Third Ring East Road, Chaoyang District; b Gatroenterology Department, Dongfang Hospital, Beijing University of Chinese Medicine, No. 6, 1st Section, Fangxingyuan, Fangzhuang, Fengtai District; c School of life sciences, Beijing University of Chinese Medicine, No. 11, North Third Ring East Road, Chaoyang District, Beijing, P. R. China.

**Keywords:** diarrhea-predominant irritable bowel syndrome, randomized controlled trial, single-cell sequencing profiling, Tong-Xie-An-Chang Decoction, traditional Chinese medicine

## Abstract

**Background:**

Diarrhea-predominant irritable bowel syndrome (IBS-D) is a kind of functional gastrointestinal disorder with obscure pathogenesis, and exploration about differential gene expression and cell heterogeneity of T lymphocytes in peripheral blood in IBS-D patients still remains unknown. Clinicians tend to use symptomatic treatment, but the efficacy is unstable and symptoms are prone to relapse. Traditional Chinese Medicine (TCM) is used frequently in IBS-D with stable and lower adverse effects. Tong-Xie-An-Chang Decoction (TXACD) has been proven to be effective in the treatment of IBS-D. However, the underlying therapeutic mechanism remains unclear. This trial aims to evaluate the clinical efficacy and safety of TXACD in IBS-D and elucidate the gene-level mechanism of IBS-D and therapeutic targets of TXACD based on single-cell sequencing technology.

**Methods/design:**

This is a randomized controlled, double-blind, double-simulation clinical trial in which 72 eligible participants with IBS-D and TCM syndrome of liver depression and spleen deficiency will be randomly allocated in the ratio of 1:1 to two groups: the experimental group and the control group. The experimental group receives Tong-Xie-An-Chang Decoction (TXACD) and Pinaverium bromide tablets placebo; the control group receives pinaverium bromide tablets and TXACD placebo. Each group will be treated for 4 weeks. The primary outcome: the rate of IBS-Symptom Severity Score (IBS-SSS). The secondary outcomes: TCM syndrome score, adequate relief and IBS-Quality of Life Questionnaire (IBS-QOL). Mechanistic outcome is the single-cell sequencing profiling of the T lymphocytes in peripheral blood from IBS-D participants before and after the treatment and healthy individuals.

**Discussion:**

This trial will prove the efficacy and safety of TXACD with high-quality evidence and provide a comprehensive perspective on the molecular mechanism of IBS-D by single-cell sequencing profiling, which makes us pinpoint specific biomarkers of IBS-D and therapeutic targets of TXACD.

## 1 Introduction

Irritable bowel syndrome (IBS) is a functional bowel disorder characterized by recurrent symptoms of abdominal pain related to defecation along with altered bowel function (i.e. changes in the form or frequency of stool), which affects around 6% of the population.^[1,2]^ IBS can further be divided into four subtypes by stool consistency, namely, constipation-predominant IBS (IBS-C), diarrhea-predominant IBS (IBS-D), or a combination of both (IBS-M).^[3]^ Among them, IBS-D has the highest clinical incidence. In the absence of reliable biomarkers, diagnosis is usually based on symptom criteria. Currently, the Rome IV Diagnostic Criteria which were published in May 2016 is generally applied for diagnosis of IBS and other FGIDs.^[4]^ IBS-D negatively impacts aspects of patients’ daily activity and social wellbeing, including work productivity, social activities, and travel.^[5]^

Thus, how to effectively treat IBS-D has become an urgent medical problem to be solved. However, the onset mechanism of IBS-D remains unclear. Numerous pathological factors are involved in the occurrence and development of IBS-D, including psychological distress,^[6]^ altered gut motility, visceral hypersensitivity,^[7]^ increased gut permeability,^[8]^ abnormal regulation of brain-gut axis,^[9]^ intestinal flora imbalance,^[10]^ immune system dysfunction and low grade inflammation.^[11,12]^ Due to the heterogeneity of the pathological process, there is no specific treatment. Hence, the therapeutic effect has not been satisfactory for patients. Currently, symptomatic treatment for IBS-D is often adopted as the treatment of consensus, such as 5-HT receptor inhibitors, Intestinal motility agents, tricyclic antidepressants, probiotics and antibiotics. Among them, pinaverium bromide tablets have frequently been used to treat IBS-related abdominal pain, defecation disorder and intestinal discomfort. But many problems still remain to be solved. The efficacy of these treatments is unstable, leading to a predisposition for IBS-D symptoms to relapse.

Chinese herbal medicine, belonging to Traditional Chinese Medicine (TCM), has been traditionally used in China and other Asian countries for thousands of years and its use is now spreading worldwide. A unique and basic feature of Chinese herbal medicine is the use of a formula containing several herbs (mixed as a cocktail) to ameliorate various abnormalities related to a certain disease.^[13]^ TCM has been shown to be an effective method for treatment of IBS-D. Currently, a series of randomized controlled trials have shown that TCM can significantly alleviate the symptoms and improve the quality of life in IBS-D patients.^[14]^ Tong-Xie-An-Chang Decoction (TXACD) is composed of seven Chinese herbs, including Baizhu (Atractylodes Macrocephala Koidz), Baishao (Paeoniae Radix Alba), Huanglian (Coptidis Rhizoma), Paojiang (Rhizoma Zingiberis Preparata), Chenpi (Citrus Reticulata), Chantui (Cicadae Periostracum), Wumei (Mume Fructus). It was created by Professor Li Junxiang from Dongfang Hospital, Beijing University of Chinese Medicine combined with his own 40 years of clinical experience, and has acquired good curative effect in the treatment of IBS-D. Previous study has shown that TXACD effectively reduced IBS-D, by relieving abdominal pain and diarrhea.^[15]^ However, TXACD therapeutic targets and molecular mechanisms directing IBS-D have not been profoundly illuminated.

About 75% of human genes can be transcribed, and transcriptome landscape is the direct reflection of encoded genetic information.^[16]^ Transcriptome profiling is able to comprehensively uncover the pathogenesis of IBS-D and the targets of TCM by analyzing the interaction networks of differentially expressed protein-coding RNAs and noncoding RNAs (ncRNAs).^[17]^ In the research of microbial ecology, cancer genome, forensic medicine, microdiagnosis, genetic imprinting and so on, the acquisition of a large number of materials has become a limiting factor for research or the data obtained from mixed materials which can’t reflect the heterogeneity of cells. Single cell sequencing solves the problem of cell heterogeneity which can’t be solved by tissue sample sequencing or when there are few samples, and provides a new direction for scientists to study and analyze the behavior, mechanism and relationship with the body of a single cell. As mentioned previously, immune system dysfunction and low grade inflammation are closely associated with the occurrence and development of IBS-D. And T lymphocytes play a key role in the pathological process. Therefore, changes in T lymphocytes transcription genes may directly affect IBS-D.

In the present trial, the primary aim of this single-center randomized controlled, double-blind, double-simulation clinical trial is to evaluate the clinical efficacy and safety of TXACD for the treatment of IBS-D, which syndrome differentiated as liver depression and spleen deficiency, and reveal the underlying mechanism of IBS-D and seek out therapeutic targets of TXACD based on single cell sequencing analysis.

## 2 Materials and methods

### 2.1 Ethical approval and consent to participate

The study is conducted in accordance with the Declaration of Helsinki (Edinburgh 2000 version). The final protocol (Date: 24 September 2020) of this trial has been approved by the Research Ethical Committee of Dongfang Hospital, Beijing University of Chinese Medicine (Version number: JDF-IRB2020032602). In addition, this trial has been registered in the Chinese Clinical Trial Registry (No. ChiCTR2000039357, registered on 24 October 2020). All participants will sign written informed consents before the enrollment. The patients will be fully informed of all contents of the study and details could be inquired in the registry web or the corresponding author. If there will be any significant modification of the protocol, it should be reviewed by the research ethical committee and updated on the registry web (http://www.chictr.org.cn) in time.

### 2.2 Study design and recruitment

**Figure 1:**
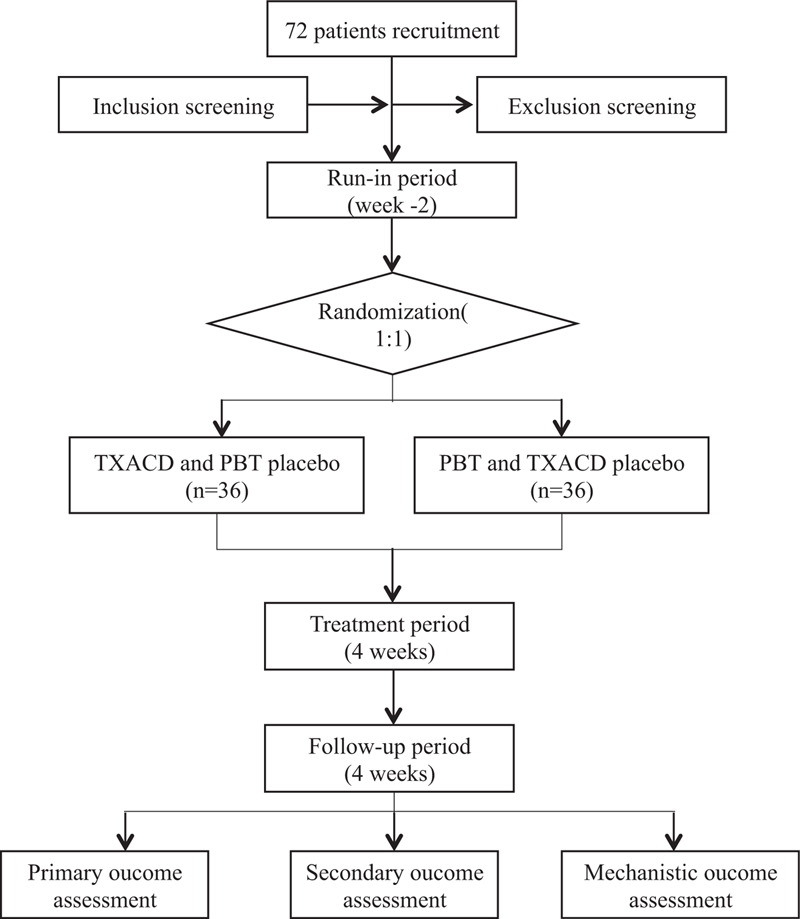
Flowchart of the trial. PBT = pinaverium bromide tablets, TXACD = Tong-Xie-An-Chang Decoction.

**Figure 2:**
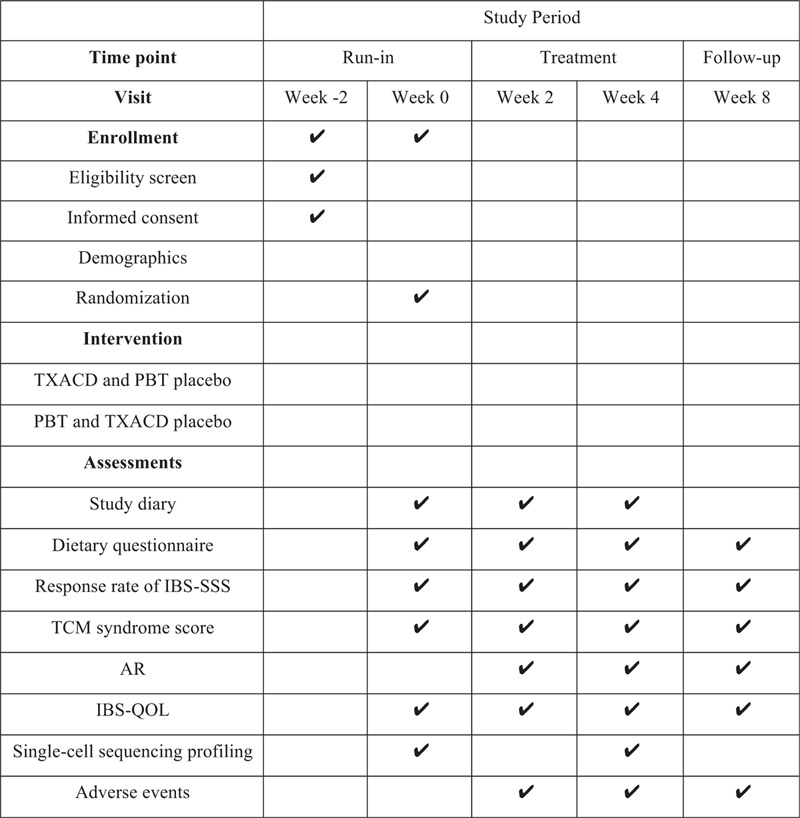
Recommended protocol items: visit schedule for enrollment, intervention, and assessments. AR = adequate relief, IBS = irritable bowel syndrome, IBS-QOL = IBS-quality of life questionnaire, IBS-SSS = IBS-symptom severity score, PBT = pinaverium bromide tablets, TCM = traditional Chinese Medicine, TXACD = Tong-Xie-An-Chang Decoction.

This clinical study is a single-center, randomized, double-blind, double-simulation trial comparing TXACD with pinaverium bromide tablets (PBT) in patients with IBS-D, which will be performed lasting totally 10 weeks. At the initial run-in period (weeks −2–0), we will assess eligibility and seek in-formed consent. Then, coming to the treatment period (weeks 0–4), eligible subjects will be randomly assigned to one of two groups (TXACD group and PBT group) in a 1:1 ratio and get blinded treatment of 4 weeks. Finally, we will have a follow-up (week 8) 4 weeks after the treatment. This study involves 5 site visits (week −2, week 0, week 2, week 4, and week 8) and 6 call visits (weekly call during the treatment period and follow-up). At end of this trial, the primary outcome and the secondary outcome will be evaluated. We will obtain peripheral venous blood from partial IBS-D patients (n = 8 for TXACD group) before and after treatment and recruited healthy controls group (n = 4) for the assessment of mechanistic outcome. The process of the study and visit schedule is schematically shown in Figures 1 and 2.

All 72 IBS-D patients participating in this study will be recruited from the gastroenterology department clinic or ward at Dongfang Hospital, Beijing University of Chinese Medicine. And we will additionally recruit 4 healthy controls.

According to the statistical minimum requirements for clinical efficacy evaluation of the two groups, the sample size of each group will be estimated to be 30 cases, with a total of 60 cases in both groups. After the shedding rate is calculated at 20%, the total number of cases in this study will be determined to be 72 cases.

### 2.3 Eligibility criteria

#### 2.3.1 Diagnostic criteria

1. The diagnostic criteria of Western medicine refer to the Rome IV consensus on diagnosis and treatment of functional gastrointestinal diseases.^[18]^
2. The TCM diagnostic criteria of the liver depression and spleen deficiency syndrome refer to consensus of TCM experts in diagnosis and treatment of Irritable Bowel Syndrome in 2017.^[19]^ TCM syndrome differentiation including the primary and secondary symptoms as following:
  - Primary symptoms:
    (1) abdominal pain accompanied by diarrhea and
    (2) prone to irascible or irritable.
  - Secondary symptoms:
    (1) distending pain in hypochondrium,
    (2) reduced appetite, and
    (3) presented with fatigue.
  - Auxiliary symptoms: light and enlarged tongue or tooth-marked tongue with white greasy coating, string pulse.

#### 2.3.2 Inclusion criteria

(1) Diagnosed with IBS-D.
(2) Agree to have a colonoscopy or provide a colonoscopy report within 3 months.
(3) The score of IBS-SSS scale was more than 75.
(4) TCM syndrome differentiation belonged to liver depression and spleen deficiency syndrome
(5) Age >18 and < 65 years.
(6) Voluntary to participate and providing written informed consents.

#### 2.3.3 Exclusion criteria

(1) Patients with organic diseases and history of abdominal surgery.
(2) Patients with contraindications for enteroscopy.
(3) Patients with severe diseases of major organs such as liver, brain, kidney, and mental disorders.
(4) Patients with systemic diseases affecting gastrointestinal motility (such as hyperthyroidism, diabetes, chronic renal insufficiency, etc).
(5) Patients are currently pregnant and lactating.
(6) Patients who had used other drugs for the treatment of irritable bowel syndrome within one week before screening.
(7) Patients with allergic constitution or have an allergy to trial medication.
(8) Patients have a history of alcohol or drug abuse.
(9) Patients have participated in other clinical trials within three months.

#### 2.3.4 Termination and withdrawal criteria

(1) The patients are unwilling to continue the clinical trial.
(2) No medication or any follow-up.
(3) Poor compliance.
(4) Serious adverse events occurred in the course of the study.
(5) The treatment plan caused obvious discomfort to the patients in the course of treatment.

### 2.4 Randomization and blindness

Institute of Clinical Pharmacology of Xiyuan Hospital, China Academy of Chinese Medical Sciences was responsible for generating the allocation sequence. Random allocation method: using SAS 9.4 (SAS Institute Inc., Cary, NC) to generate random sequences, randomly grouping, and coding drugs; Concealment and implementation of allocation scheme: the secret envelope method was used to conceal the random sequences.

The double-blind trial blinded the implementers of the study (doctors) and the subjects (patients). In order to avoid the impact of blindness on other research subjects due to serious adverse conditions, only a number is given to each study object, and the medicine is distributed according to the number. Doctors and patients can only have access to the blind number in this process, and the blind bottom is kept by a person who will not participate in this study. In the event of an emergency or when the patient needs to be rescued, it is necessary to know what kind of treatment the patient is receiving, the corresponding emergency letter shall be opened in the presence of at least two researchers, and the emergency letter shall be collected together with the case report form after the end of the trial. Blind bottom leakage or emergency letter reading should not exceed 20% before the end of the trial.

### 2.5 Research person

Experienced physicians, as investigators, will take direct charge of recruiting and evaluating participants in this trial. Postgraduates in gastroenterology will be responsible for dispatching drugs, filling in case report forms, and recording detailed reasons for the participants’ withdrawal from the trial. And they will be trained by the principal investigator before participating in this trial.

### 2.6 Recruitment and encouragement

A total 72 patients will be recruited from the gastroenterology department clinic or ward at Dongfang Hospital, Beijing University of Chinese Medicine, from January 2020 until December 2022. Before the recruitment, all the participants will be fully informed about consent of this trial, including therapeutic interventions, trial benefits, and possible risks. Participants could be enrolled in it only after signing their written informed consent. Meanwhile, in order to guarantee the completeness of this trial, we will handsel drugs to the patients who participate it completely.

### 2.7 Collection of biological specimens

The participants’ blood, stool and urine samples will be collected before and after intervention, and clearly marked in a uniform format. A designated investigator will isolate T lymphocytes single cells from the blood samples in the laboratory. Then, the isolated single cells were put into 3.5 μ l lysate and repeatedly blown with a pipette for several times, and then immediately stored at -80°C, finally transported to Shanghai OE Biotech Co., Ltd (Shanghai, China) for transcriptome sequencing.

## 3 Intervention

### 3.1 Medications

TXACD (TXACD granules supplied by Beijing Tcmages Pharmaceutical Co., LTD, Beijing, China), pinaverium bromide tablets (PBT, 50 mg/tablet, supplied by Beijing Wansheng Pharmaceutical Co. LTD, Beijing, China), and placebos are prepared respectively in accordance with the 2 above drugs in terms of appearance, smell, taste, packaging, label and other characteristics (TXACD granule placebo is supplied by Beijing Tcmages Pharmaceutical Co., LTD, Beijing, China; PBT placebo is supplied by Beijing Wansheng Pharmaceutical Co. LTD, Beijing, China). TXACD is composed of seven Chinese herbs, including Baizhu (Atractylodes Macrocephala Koidz), Baishao (Paeoniae Radix Alba), Huanglian (Coptidis Rhizoma), Paojiang (Rhizoma Zingiberis Preparata), Chenpi (Citrus Reticulata), Chantui (Cicadae Periostracum), Wumei (Mume Fructus). In this trial, an administrator is assigned to manage the drugs, including drug storage, distribution, and recycling and record the detailed information timely.

### 3.2 Intervention schedule

TXACD granule or placebo will be orally administered one packet after dissolved twice a day (1 h after the meal) for 4 weeks. Pinaverium bromide tablets or placebo will be administered one tablet three times a day (swallowed with meals). During the study, participants will be asked to stop taking the medications associated with IBS-D. Probiotics, functional foods and dietary supplements are also banned. Participants will be obligated to fill out daily study diaries, including medication administration records, accompanying treatments, and dietary questionnaires to help check compliance and statistically correct dietary bias. The presence of worsening IBS-D symptoms, which require recording in the Case Report Form (CRF), allows rescue medication to be used for less than 1 week.

### 3.3 Follow-up

Each participant will have a participant diary card recording signs and symptoms of abdominal pain and fecal character. After enrolling in the trial, participants will be interviewed every two weeks, and they should return any remaining medication and packaging, and receive medications they need to take for the next two weeks. The evaluation of study endpoints and follow-up duration is shown in Figure 2.

### 3.4 Outcome measurements

#### 3.4.1 Primary outcome

The primary outcome of our trial is the response rate to the IBS Symptom Severity Score (IBS-SSS).^[20]^ Respondents were defined as having 50% or more reduction in IBS symptoms compared to baseline. IBS-SSS will be evaluated at baseline, week 2, week 4 and week 8.

#### 3.4.2 Secondary outcome

The secondary outcomes including TCM syndrome score, adequate relief (AR) and IBS-Quality of Life Questionnaire (IBS-QOL).

#### 3.4.3 TCM syndrome score

According to the diagnostic criteria of liver depression and spleen deficiency syndrome, the main symptoms, secondary symptoms will be graded and scored. All the indexes will be recorded at baseline, week 2, week 4 and week 8.

According to the referred Guidance Principle of Clinical Study on New Drug of Traditional Chinese Medicine,^[21]^ All the symptoms will be divided into four grades: none, mild, moderate and severe, with 0, 2, 4 and 6 points in the main symptoms; 0, 1, 2 and 3 points in the secondary symptoms. The tongue and pulse manifestations will be divided into normal and abnormal grades, 0 and 2 points in the main symptoms, and 0 and 1 points in the secondary symptoms.

The clinical efficacy is evaluated as follows:

(1) Clinical recovery: clinical symptoms disappeared or syndrome score decreased by ≥95% from the baseline.
(2) Marked efficacy: the syndrome score decreased by ≥70% and <95% compared with the baseline.
(3) Efficacy: the syndrome score was a decrease of at least 30% and less than 70% from the baseline.
(4) Invalid: the syndrome score was a decrease of less than 30% from the baseline.
(5) Worsen: the syndrome score exceeded the baseline after treatment.
(6) TCM syndrome efficacy rate = (Clinical recovery + Marked efficacy + Efficacy) cases /a total number of cases × 100%.

#### 3.4.4 AR

Participants will receive 8 telephone interviews (weeks 1 to 8) and will be asked the following questions: “Have you had adequate relief from your IBS pain and discomfort in the past week?” Participants simply need to answer “yes” or “no”. Respondents are defined as having answered “yes” at least 4 of the 8 weeks. AR will be evaluated at week 8.

#### 3.4.5 Irritable Bowel Syndrome-Quality of Life Questionnaire (IBS-QOL)

IBS-QOL which reflects a combination of physical and mental health across eight dimensions, will be assessed at baseline, week 2, week 4 and week 8.

#### 3.4.6 Mechanistic outcome

We will analyze the single-cell sequencing profiling of the T lymphocytes in peripheral blood from the TXACD granule group (n = 8) at baseline and week 4, and healthy subjects (n = 4) at baseline. In IBS-D, a complete set of transcriptional deviations enables us to search for potential molecular mechanisms and specific diagnostic biomarkers. At the same time, the therapeutic targets and pathways of TXACD in IBS-D were clarified. On the Illumina HiSeq 2000 platform, the preparation and deep sequencing of the transcriptome libraries will be performed by Shanghai OE Biotech Co., Ltd (Shanghai, China).

### 3.5 Safety assessment

Participants will undergo laboratory tests at baseline and week 4, including liver and kidney function (ALT, AST, Scr, BUN), blood routine test, urine routine test, and fecal occult blood tests. Other tests include an electrocardiograph examination.

Adverse events (AEs) will be defined in the whole trial process by TXACD granule, TXACD granule placebo, PBT or PBT placebo caused by any unpredictable and unexpected harmful effects. A research assistant will record the detailed information including symptoms, signs, severity, start date, duration, lab results, intervention, and the results of adverse events at every visit. Once severe adverse events (SAEs) occur, the event must be reported to the principal investigator, the hospital ethics Committee and the Beijing Food and Drug Administration within 24 hours, and the participants’ safety should be the top priority. The principal investigator has the power to terminate the trial if necessary.

### 3.6 Data management and quality control

All records will be collected by trained and qualified investigators and screened by a qualified investigator. Once the CRF is complete, the original record will not be changed, even if any modifications are made. The completed CRFs will be reviewed by a clinical inspector. Data entry and management will be guided by medical statisticians. To ensure the accuracy of the data, 2 data managers will input and proofread the data independently. Once the created database has been audited and verified to be correct, the data will be locked by the principal investigator and statistical experts. The locked database or files will not be changed and will be submitted to the research team for statistical analysis. In addition, all data sets will be managed and monitored by Guangzhou Hipower Pharmaceutical R&D Co., Ltd. It will audit trials and data stores on a weekly basis.

### 3.7 Statistical analysis

#### 3.7.1 Clinical data analysis

Data analysis will be performed by professional statisticians and principal investigator using IBM SPSS Statistics (Version 24.0 position IBM Corp., Armonk, NY). Statistical analysis included actual number of subjects selected, cases of detachment and rejection, demographic and other baseline characteristics, patient compliance, efficacy, and safety analysis. Categorical variables will be described by frequency tables or percentages and continuous variables will be described by mean ± standard deviation. Categorical data will be analyzed by chi-square test or Fisher exact test to compare the differences between the two groups. Besides, quantitative data with normal distribution will be analyzed by T test. If the data does not conform to normal distribution or variance uniformity, Wilcoxon rank sum test or Wilcoxon signed test will be used for comparison between the two groups. A 2-sided *P* < .05 indicates statistical significance. For missing data, sensitivity analysis will be conducted and the optimal approach to the imputation of missing data will be proposed.

#### 3.7.2 Mechanistic data analysis

The differential expression analysis about mRNA for all pairwise comparisons: IBS-D patients versus healthy controls, TXACD (pre-treatment) versus TXACD (post-treatment), will be performed by Cuffdiff. An adjusted *P* < .05 (Student *t* test accompanied with Benjamini-Hochberg FDR adjustment) will be used as the cut-off point for significantly differentially expressed genes. Prediction of target genes will be performed using Pearson correlation test.

## 4 Discussion

Diarrhea-predominant irritable bowel syndrome (IBS-D) is a chronic intestinal disease,^[22]^ which seriously affects the study and life of the patients. However, little is known about the pathogenesis of IBS-D. Therefore, there is no specific drug in treating IBS-D. Clinicians are accustomed to using symptomatic treatment, but the effect remains erratic.

At present, more attention has been focused on Traditional Chinese Medicine (TCM) with its well-defined and established therapeutic system.^[23]^ TCM, with traditional Chinese syndrome differentiation as the core, has a stable effect in the treatment of IBS-D. Tong-Xie-An-Chang Decoction (TXACD), created by professor Jun-xiang Li from Dongfang Hospital, Beijing University of Chinese Medicine, has been shown to be effective in relieving abdominal pain in IBS-D patients in our previous randomized positive controlled clinical trial.^[15]^ Simultaneously, in IBS-D experimental rat model, TXACD was proved that it could reduce the fecal water content and abdominal withdrawal reflex (AWR) score, used to evaluate visceral sensitivity.^[24]^ These results of clinical and animal experiments suggested that TXACD might be a novel and promising strategy for the treatment of IBS-D. However, the potential therapeutic mechanism of TXACD in treating IBS-D still remains to be uncovered.

Although IBS-D is traditionally considered to be a functional bowel disease, many studies have shown that patients with IBS-D have low-grade inflammation and abnormal T cell activation on microscopic and molecular levels.^[25–27]^ Nevertheless, most of these studies are based on tissue, cell population level and expressed cytokines. It is not clear what the difference, cell heterogeneity, is at the single cell level. Single-cell RNA-sequencing technology may help elucidate this possibility of understanding cell heterogeneity. In the present study, Single-cell RNA-sequencing analysis is used to evaluate the transcriptome changes of T lymphocytes in peripheral blood of patients so as to explore the mechanism of T lymphocyte heterogeneity changes in the pathogenesis of IBS-D using differential gene analysis and cellular heterogeneity analysis and reveal the mechanism of TXACD in treating IBS-D at single cell level.

Because we did not use the blind method, the placebo effect might exist in our previous clinical studies, which may lead to defects in the evaluation of clinical effects of TXACD in treating. Therefore, we will adopt the research method of randomized controlled trial to conduct the present clinical study to evaluate the efficacy and safety, and explore the underlying mechanism of TXACD in treatment of IBS-D.

To sum up, we hope that this trial will provide high-quality evidence for the efficacy and safety of TXACD. We also expect to have a deeper understanding of the pathogenesis of IBS-D, identify specific biomarkers, and accurately locate the target of TXACD in treating IBS-D.

## Acknowledgments

We are grateful to all the researchers and patients who have been involved in this trial.

## Author contributions

**Conceptualization:** Jun-xiang Li, Xiao-xuan Xue.

**Investigation:** Lei Shi, Ya-li Yuan.

**Supervision:** Chun-e Xie, Wen-jing Pei.

**Writing – original draft:** Xiang Tan, Xing-jie Zhao.

**Writing – review & editing:** Xiao-xuan Xue.
